# Influence of Chronic Fatigue Syndrome Codiagnosis on the Relationship between Perceived and Objective Psychoneuro-Immunoendocrine Disorders in Women with Fibromyalgia

**DOI:** 10.3390/biomedicines11051488

**Published:** 2023-05-20

**Authors:** Eduardo Otero, Isabel Gálvez, Eduardo Ortega, María Dolores Hinchado

**Affiliations:** 1 Immunophysiology Research Group, Instituto Universitario de Investigación Biosanitaria de Extremadura (INUBE), 06080 Badajoz, Spain; eoteroc@unex.es (E.O.); igalvez@unex.es (I.G.); mhinsan@unex.es (M.D.H.); 2 Immunophysiology Research Group, Physiology Department, Faculty of Sciences, University of Extremadura, 06071 Badajoz, Spain; 3 Immunophysiology Research Group, Nursing Department, Faculty of Medicine and Health Sciences, University of Extremadura, 06071 Badajoz, Spain

**Keywords:** fibromyalgia, chronic fatigue syndrome, accelerometry, inflammation, stress, cortisol, IL-8, catecholamines, serotonin, oxytocin

## Abstract

Although the predominant symptom in fibromyalgia (FM) is muscle pain, and fatigue in chronic fatigue syndrome (CFS), differential diagnosis is very difficult. This research investigates the psychoneuroimmunoendocrine disorders of FM patients and ascertains whether a previous CFS diagnosis affected them. Through accelerometry objective parameters, physical activity/sedentarism levels in relation to fatigue are studied, as well as whether perceived levels of stress, anxiety, and pain correspond to objective biomarkers, all of these with respect to a reference group (RG) of women without FM. FM patients have a worse psychological state and perceived quality of life than those with RG. These perceived outcomes are consistent with impaired objective levels of a sedentary lifestyle, higher systemic levels of cortisol and noradrenaline, and lower levels of serotonin. However, FM patients with a previous CFS diagnosis had lower systemic levels of IL-8, cortisol, oxytocin, and higher levels of adrenaline and serotonin than FM patients without diagnosed CFS. In conclusion, while perceived health parameters do not detect differences, when objective neuroimmunoendocrine parameters related to stress, inflammation, pain, and fatigue are used, people with CFS could be overdiagnosed with FM. This reinforces the need for objective biomarker assessment of these patients for better diagnostic discrimination between both syndromes.

## 1. Introduction

Fibromyalgia (FM) is defined as a syndrome characterized by chronic widespread pain associated with other physical disorders such as hyperalgesia, allodynia, and fatigue [1]. Prevalence of FM is approximately 6.3%, and it is considerably more frequent in women (90% of cases), according to the World Fibromyalgia Association. Differential diagnosis of FM is a serious problem, because it is often associated with different pathologies that present similar symptoms, both perceived symptoms and those assessed through objective biomarkers. One of the most frequently associated syndromes is chronic fatigue syndrome (CFS), defined as disabling fatigue of 6 months or more duration, which is also related to other disorders such as psychological disturbances and unrefreshing sleep [2].

Although the aetiology of FM is not completely established, both abnormalities in the function of the autonomic nervous system and neuroimmunoendocrine alterations have been implicated in its pathogenesis. There are many studies through which our research group has established a clear relationship between FM and dysregulation of the hypothalamic-pituitary-adrenal (HPA) axis [3,4,5]. This alteration strongly contributes to persistent pain and affective distress; it may be mediated by pro-inflammatory cytokines, chemokines, and stress mediators, such as systemic IL-8 and cortisol [3,6,7,8,9]. Additionally, in CFS, neuroimmunoendocrine disruption has been reported [10] and potential biomarkers for CFS diagnosis have been reported [11]; however, their validation has been difficult [12,13]. There are also controversies in the possible imbalance of inflammatory cytokines in the case of CFS, where distinctly different cytokine association networks have been reported in healthy individuals [11].

In this context, although both syndromes have a high percentage of comorbidity, limited studies have considered CFS codiagnosis and how it influences quality of life, objective physical activity and fatigue, and the neuroimmunoendocrine status of patients with FM. Therefore, based on the need to find objective biomarkers reflecting the symptoms and perceived health of patients with FM that could help to make a differential diagnosis between the two syndromes, we hypothesized that CFS codiagnosis would negatively influence physical activity and the already dysregulated neuroimmunoendocrine status of patients with FM. The overall aim was to delve into the psychoneuroimmunoendocrine disorders of patients with FM and to test whether a previous CFS diagnosis could affect them, assessing both patients’ perception through scientifically validated questionnaires, and objectively through accelerometry and systemic immunophysiological biomarkers of inflammation, stress, and anxiety.

This differential study is justified in the context of the decreased capacity for daily activity that these patients present due to pain and other nervous disorders. We believe that this research could contribute to validating and objectifying the more subjective aspects of patients’ quality of life, through objective biomarkers, and thus improve the differential diagnosis of these syndromes.

## 2. Materials and Methods

### 2.1. Participants and Experimental Design

This study was carried out with 34 patients (total group of FM patients), all aged between 40 and 65 years. They belonged to the FM associations of Extremadura, an autonomous community whose population is very homogeneous in terms of lifestyle. The majority of the population are covered by the Spanish National Health System. Moreover, this region is a reference region in health research in Spain [14,15], particularly for the study of fibromyalgia and of the effects of exercise internationally [16]. A total of 17 of these FM patients had a previous CFS diagnosis (FM + CFS group) and the remaining 17 were FM patients without a CFS codiagnosis (FM group). A group of 11 women of the same age range constituted the reference group of “healthy” women not diagnosed with FM, CFS or any other inflammatory or rheumatic pathology, or any condition involving depression, anxiety and/or pain (RG group).

The selected patients met the following inclusion criteria: (a) FM diagnosis with or without a previous CFS codiagnosis by rheumatologists or internal medicine professionals according to the American College of Rheumatology (ACR) diagnostic criteria for FM patients [1], and the Fukuda and co-workers criteria for CFS patients [2]; (b) age 40 to 65 years; (c) not having a diagnosis of depression; (d) not having multiple chemical sensitivity; (e) not having performed scheduled physical activity in the previous two months or during the accelerometry tests; (f) not taking corticosteroids or anti-cytokine therapy.

At the first phase of the study, anthropometric characteristics, employment status and body composition were assessed (Table 1). Medication history was very diverse in each patient; however, the vast majority of women had a prescription for different types of anti-inflammatory and analgesic drugs (e.g., ibuprofen, dexketoprofen, paracetamol, tramadol), excluding patients with prescribed corticosteroid treatments or anti-cytokine therapies.

Subsequently, each participant completed the questionnaires given to them individually, in a supervised manner and with the corresponding indications as to how and when to complete them. These questionnaires were finally collected for the quantification of the values and their subsequent analysis. Afterwards, each participant was given an accelerometer, which enables the objective assessment of physical activity and sedentary lifestyle. All volunteers were required to wear it on the wrist of their non-dominant hand. In order to obtain more information and given that the pace of life varies depending on whether it is a weekend or a weekday, the study was conducted over seven days. After the last day, the accelerometers were collected for further processing of the recorded data using the “Actilife v.6” software (ActiGraph, LLC., Pensacola, FL, USA), and blood samples were taken for determination of neuroimmunoendocrine biomarkers by ELISA.

### 2.2. Body Composition Measurements; Bioimpedance Analysis

The BIA TANITA DC-360 digital scale (manufactured by Tanita in Tokyo, Japan) was employed to evaluate body composition. The scale’s measurement frequencies ranged from 6.25 kHz to 50 kHz and yielded data on several parameters including body fat mass (%), bone mass (kg), muscle mass (kg), body water (%) and visceral fat index. The BMI was computed using the weight/height formula expressed in kg/m^2^. The participants were assessed while fasting, wearing light clothing, and with bare feet.

### 2.3. Subjective Quality of Life

The Spanish version of Beck’s Depression Inventory developed by Sanz and co-workers [17] was used to determine the presence of signs of depression. Higher scores are associated with greater signs of depression and according to the final score, perceived depression can be classified as: mild (10–19), moderate (20–30), or severe (>30) [18].

The Spanish version of the Perceived Stress Scale (PSS) developed by Remor was used to determine perceived stress levels. It is composed of 14 items with a Likert scale response format, where a higher total score corresponds to a higher level of perceived stress [19,20].

The State-Trait Anxiety Inventory (STAI) is composed of two subscales: state anxiety (transient emotional condition) and trait anxiety (relatively stable characteristic of anxiety proneness) [21]. Each subscale is composed of a total of 20 items in a Likert-type response system. As in the previous questionnaires, higher scores indicate a higher anxiety state. In the present study, the Spanish version of Buela-Casal and Guillén-Riquelme [22] was used.

The Brief Pain Inventory (BPI) and the Brief Fatigue Inventory (BFI) are two self-administered questionnaires designed to assess perceived pain and fatigue, respectively [23,24]. Both consist of two basic magnitudes: intensity and interference scored on scales from 0 “no pain/fatigue” to 10 “worst pain/fatigue”. Higher scores correlate directly with a higher perception of pain and fatigue. The Spanish version of the BPI by Badía and co-workers [25] and the Spanish version of the BFI by Valenzuela and co-workers [26] were used.

In order to detect and quantify lifestyle patterns that reflect health improvement and adequate life control, the Healthy Lifestyle and Personal Control Questionnaire (HLPCQ) was used. This questionnaire is composed of several items: choice of a healthy diet, avoidance of a harmful diet, daily routine, organized physical exercise and social and mental balance [27].

The Spanish version of the Fibromyalgia Impact Questionnaire (FIQ) by Rivera and González [28] was used to assess the impact of FM on physical functioning, the ability to perform usual work and the degree to which FM has affected this activity, as well as subjective items closely related to the clinical profile of FM (pain, fatigue, tiredness and stiffness) and emotional state (anxiety and depression) [29]. To obtain the total score, the different items were normalized; therefore, the total score ranged from 0–80 [28]. A higher score indicates a negative impact of FM on the patient’s health.

Finally, questionnaires aimed at assessing fear and anxiety about Coronavirus Disease 2019 (COVID-19): Coronavirus Anxiety Scale (CAS) and COVID-19 Fear Scale-19 (FCV-19S) were included. The CAS is a brief mental health assessment that can identify cases of dysfunctional anxiety related to COVID-19 [30]. The Spanish version of Caycho-Rodriguez and co-workers was used [31]. The FCV-19S identifies individuals with high levels of fear of COVID-19 [32]. In the present study, the Spanish version of Sánchez-Teruel and Robles Bello was used [33]. In both questionnaires, a higher score is interpreted as higher anxiety and fear of COVID-19.

### 2.4. Determination of Objective Levels of Physical Activity and Sedentary Lifestyle

The Actigraph Wgt3x—BT accelerometer was used for the objective determination of physical activity/sedentary levels. This model records the change in acceleration of the center of mass in three planes of motion (x, y, z) and converts them into a quantifiable digital signal called counts. Therefore, counts are units of motion, and each count record is summed and stored in the accelerometer’s memory in a configurable period called epoch [34]. In our study, we used an epoch of one minute.

Subsequently, the following parameters were analyzed: count, maximum and average duration of activity and sedentary bouts, and prediction of the metabolic rate through the METs (Metabolic Equivalent of Task) using the algorithm established by Freedson and co-workers [35] through the “Actilife” software (ActiGraph, LLC, Pensacola, FL, USA).

### 2.5. Blood Collection and Serum Isolation

On the same day of the actigraphic device collection, blood samples were collected from fasting subjects at 08:00 and placed in collection tubes for serum isolation, where they were kept for 15–20 min at room temperature. The serum was centrifuged at 1800× *g* for 15 min. Serum samples were gradually refrigerated at −20 °C as they were obtained. Finally, the samples were stored at −80 °C until further analysis.

### 2.6. Determination of Neuroimmunoendocrine Markers

Serum concentrations of cortisol (DetectX^®^, ArborAssays, Ann Arbor, MI, USA), dehydroepiandrosterone (DHEA) (Demeditec Diagnostic GmbH, Kiel, Germany), serotonin (Reddot Biotech. Inc. Katy, TX, USA), oxytocin (CloudClone Corp. Katy, TX, USA), adrenaline and noradrenaline (Demeditec Diagnostic GmbH, Kiel, Germany), interleukin-8 (IL-8) and interleukin-10 (IL-10) (Diaclone SAS, Biotech. Inv. Group, Besancon Cedex, France) were measured using commercial ELISA kits.

### 2.7. Statistical Analysis

The values are expressed as mean ± SEM. Normality of the variables was checked via the Shapiro–Wilk test, followed by Student’s *t*-test for normally distributed samples or Mann–Whitney test for nonparametric samples. Chi-square independence test was used for comparisons between qualitative variables expressed as a percentage. The minimum level of significance was set at *p* < 0.05. Statistical analysis was performed with the SPSS^®^ Statistics v.27.0 package (IBM Corp., Armonk, NY, USA).

## 3. Results

Firstly, as stated in Table 1, all participants were white women and had been diagnosed with FM (with or without previous CFS diagnosis) for more than two years. Regarding work status, we can see that there were no significant differences between any of the experimental groups. No significant differences were found in age, BMI, fat mass (%), bone mass (kg), body water (%), muscle mass (kg). However, FM patients both with and without CFS codiagnosis had a significantly higher visceral fat index compared to the reference group (*p* < 0.05).

### 3.1. Psychological Status and Quality of Life

Table 2 shows the psychological status and quality of life. The total group of FM patients showed worse levels (*p* < 0.001) of depression, stress, anxiety, pain, fatigue, impact of fibromyalgia, as well as worse levels (*p* < 0.01) of anxiety and fear towards COVID-19, with respect to the reference group. We can also observe that CFS codiagnosis does not affect FM patients’ already-impaired psychological state and perceived quality of life. Higher values (*p* < 0.05) for perceived fatigue were found in FM patients with a previous CFS diagnosis.

### 3.2. Neuroimmunoendocrine Biomarkers

Table 3 shows the systemic concentrations of neuroimmunoendocrine biomarkers in the total group of FM patients compared to the reference group. No significant differences were found in serum levels of IL-8, IL-10 (represented as the percentage of the values above the Lower Limit of Detection (LLD)), DHEA, adrenaline, and oxytocin in total FM patients with respect to the reference group. Nevertheless, higher levels of cortisol (*p* < 0.05), noradrenaline (*p* < 0.05), and lower levels of serotonin (*p* < 0.05) were found in total FM patients.

#### 3.2.1. Influence of CFS Codiagnosis in FM Patients: Serum Levels of IL-8 and IL-10

**Figure 1:**
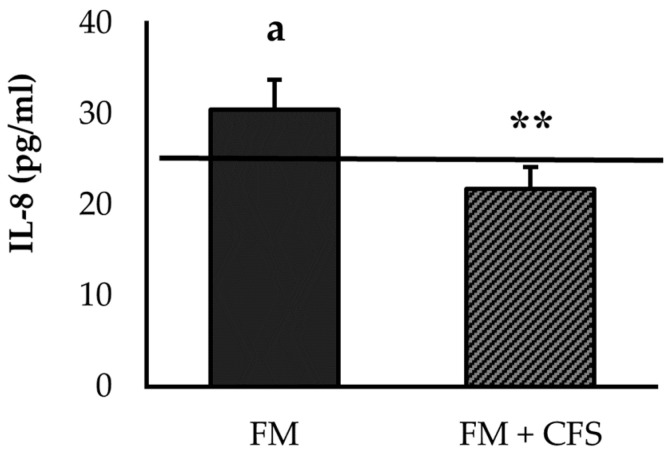
Serum levels of IL-8 in FM patients with (FM + CFS, *n* = 17) or without (FM, *n* = 17) CFS diagnosis. The horizontal line represents values obtained in the age-matched reference group of “healthy” women. Columns represent the mean ± SEM of independent assays performed in duplicate for each participant. IL-8: Interleukine-8, FM: Fibromyalgia, CFS: Chronic Fatigue Syndrome. ** *p* < 0.01 with respect to FM group. ^a^ *p* < 0.05 with respect to the reference group. (Student’s *t*-test).

FM patients with a previous CFS diagnosis had a significantly lower concentration of IL-8 than patients without CFS (*p* < 0.01; Figure 1). No significant differences in serum levels of IL-8 were found between FM patients with a previous CFS diagnosis and the reference group. However, FM patients without CFS presented higher serum IL-8 levels compared to the reference group (*p* < 0.05), which were also above the reference value (>29 pg/mL) obtained in numerous studies in FM patients of our research group [3,4,5].

As explained previously, the results related to IL-10 were determined as the percentage of patients with serum levels of IL-10 above the LLD. No significant differences were found between both experimental groups (13.3% > LLD in FM patients versus 17.6% > LLD in FM patients with codiagnosis of CFS), and with respect to the reference group.

#### 3.2.2. Influence of CFS Codiagnosis in FM Patients: Serum Levels of Cortisol and DHEA

**Figure 2:**
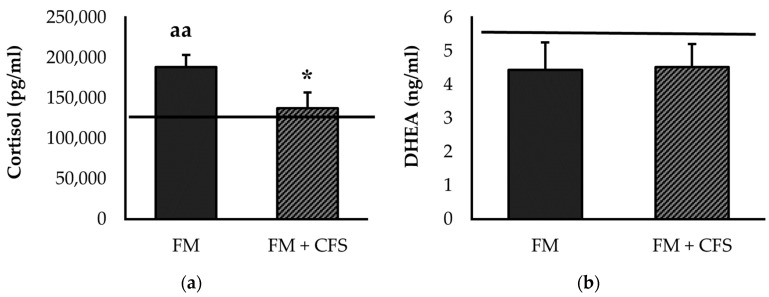
Serum levels of cortisol (**a**) and DHEA (**b**) in FM patients with (FM + CFS, *n* = 17) or without (FM, *n* = 17) CFS diagnosis. The horizontal line represents values obtained in the age-matched reference group of “healthy” women. Columns represent the mean ± SEM of independent assays performed in duplicate for each participant. DHEA: Dehydroepiandrosterone, FM: Fibromyalgia, CFS: Chronic Fatigue Syndrome. * *p* < 0.05 with respect to FM group. ^aa^ *p* < 0.01 with respect to the reference group. (Mann–Whitney *u*-test).

Figure 2 represents the systemic concentrations of stress-related hormones cortisol (Figure 2a) and DHEA (Figure 2b). Significantly lower serum levels of cortisol were found in FM patients with previous CFS diagnosis compared to FM patients without CFS (*p* < 0.05). Compared to the reference group, no significant differences in serum levels of cortisol were found in FM patients with a previous CFS diagnosis; however, significantly higher serum levels of cortisol were found in FM patients without a CFS diagnosis (*p* < 0.01). Regarding serum levels of DHEA, no significant differences were found in FM patients with or without CFS codiagnosis. No significant differences were found in either experimental group with respect to the reference group.

#### 3.2.3. Influence of CFS Codiagnosis in FM Patients: Serum Levels of Noradrenaline and Adrenaline

**Figure 3:**
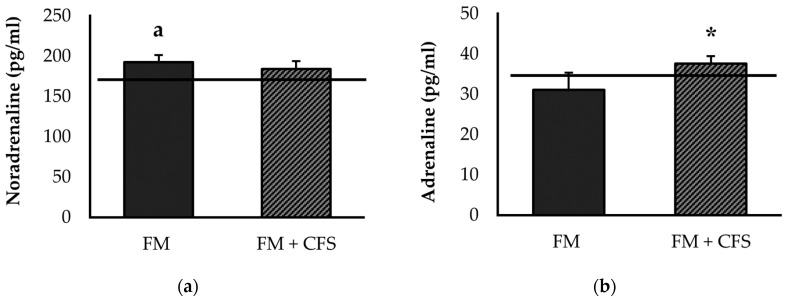
Serum levels of noradrenaline (**a**) and adrenaline (**b**) in FM patients with (FM + CFS, *n* = 17) or without (FM, *n* = 17) CFS diagnosis. The horizontal line represents values obtained in the age-matched reference group of “healthy” women. Columns represent the mean ± SEM of independent assays performed in duplicate for each participant. FM: Fibromyalgia, CFS: Chronic Fatigue Syndrome. * *p* < 0.05 with respect to FM group. ^a^ *p* < 0.05 with respect to the reference group. (Student’s *t*-test).

Figure 3 shows serum concentrations of noradrenaline (Figure 3a) and adrenaline (Figure 3b). No significant differences were found in serum levels of noradrenaline between FM patients with or without CFS codiagnosis. However, significantly higher serum concentrations of noradrenaline were found only in FM patients without a CFS codiagnosis with respect to the reference group (*p* < 0.05).

On the other hand, FM patients with CFS codiagnosis had significantly higher serum adrenaline values compared to FM patients without CFS (v < 0.05). No significant differences were found in the serum levels of adrenaline in FM patients with or without CFS with respect to the reference group.

#### 3.2.4. Influence of CFS Codiagnosis in FM Patients: Serum Levels of Serotonin and Oxytocin

**Figure 4:**
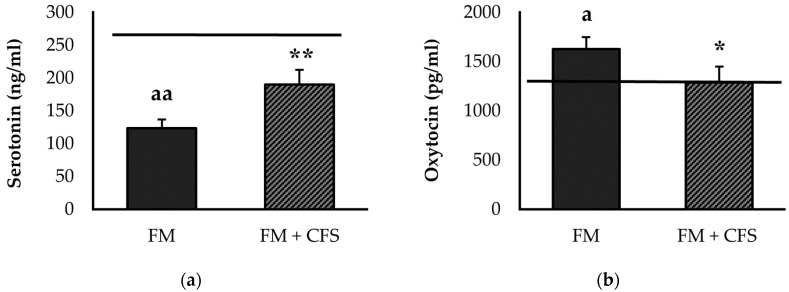
Serum levels of serotonin (**a**) and oxytocin (**b**) in FM patients with (FM + CFS, *n* = 17) or without (FM, *n* = 17) CFS diagnosis. The horizontal line represents values obtained in the age-matched reference group of “healthy” women. Columns represent the mean ± SEM of independent assays performed in duplicate for each participant. FM: Fibromyalgia, CFS: Chronic Fatigue Syndrome. * *p* < 0.05, ** *p* < 0.01, with respect to FM group. ^a^ *p* < 0.05, ^aa^ *p* < 0.01 with respect to the reference group. (Student’s *t*-test).

Serum levels of serotonin (Figure 4a) and oxytocin (Figure 4b) are depicted in Figure 4. FM patients with CFS codiagnosis presented significantly higher serum levels of serotonin than FM patients without CFS (*p* < 0.01). In contrast, patients with CFS codiagnosis showed serum serotonin levels that were very close to those of our reference group, without significant differences. However, FM patients without CFS codiagnosis showed significantly lower serum levels of serotonin with respect to the reference group (*p* < 0.01).

In addition, significantly lower serum oxytocin values were found in FM patients with a previous CFS diagnosis compared to patients without CFS diagnosis (*p* < 0.05). No significant differences were found in serum levels of oxytocin in FM patients with CFS codiagnosis with respect to the reference group. Nevertheless, FM patients without CFS diagnosis had higher serum levels of oxytocin compared to the reference group (*p* < 0.01).

### 3.3. Physical Activity/Sedentarism Levels Determined via Accelerometry

Table 4 shows physical activity/sedentarism parameters determined via accelerometry in the total group of patients with FM compared to the reference group. Although no significant differences were found in caloric expenditure (METs), the total group of FM patients showed lower activity bouts (*p* < 0.05) and shorter time of activity bouts, both total time (*p* < 0.05) and average time (*p* < 0.05), as well as lower step counts (*p* < 0.01) with respect to the reference group. Related to sedentary parameters, total FM patients presented higher number of sedentary bouts (although without significant differences) and longer total sedentary time (*p* < 0.01) with respect to the reference group.

#### 3.3.1. Influence of CFS Codiagnosis in FM Patients: Accelerometric Study of Physical Activity

**Figure 5:**
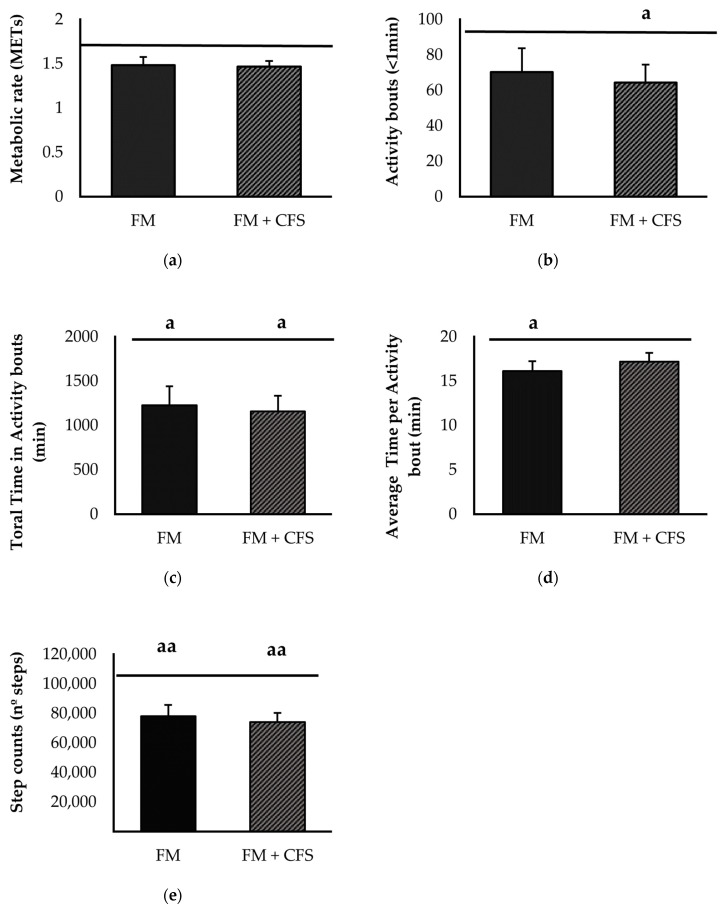
Physical activity levels determined via accelerometry: Metabolic rate (**a**) activity bouts (**b**), total time in activity bouts (**c**), average time per activity bout (**d**) and steps counts (**e**) in FM patients with (FM + CFS, *n* = 17) or without (FM, *n* = 17) CFS diagnosis. The horizontal line represents values obtained in the age-matched reference group of “healthy” women. Columns represent the mean ± SEM of independent assays performed in duplicate for each participant. FM: Fibromyalgia, CFS: Chronic Fatigue Syndrome, MET: Metabolic Equivalent of Task. ^a^ *p* < 0.05, ^aa^ *p* < 0.01 with respect to the reference group. (Student’s *t*-test).

Figure 5 represents physical activity parameters measured via accelerometry in FM patients with and without previous CFS diagnosis, separately. No significant differences were found between FM patients with and without CFS diagnosis in any of the parameters studied: METs (Figure 5a), activity bouts (Figure 5b), total duration of activity bouts (Figure 5c), average duration of activity bouts (Figure 5d) and step count (Figure 5e). However, as expected, both experimental groups showed, in general, lower values of objective physical activity than the reference group; FM patients without CFS codiagnosis presented significantly lower total and average times of activity bouts (*p* < 0.05 in both) and step counts (*p* < 0.01). FM patients with a previous CFS diagnosis presented lower total time and total activity bout counts (*p* < 0.05 in both) and lower step counts (*p* < 0.01).

#### 3.3.2. Influence of CFS Codiagnosis in FM Patients: Accelerometric Study of Sedentarism

**Figure 6:**
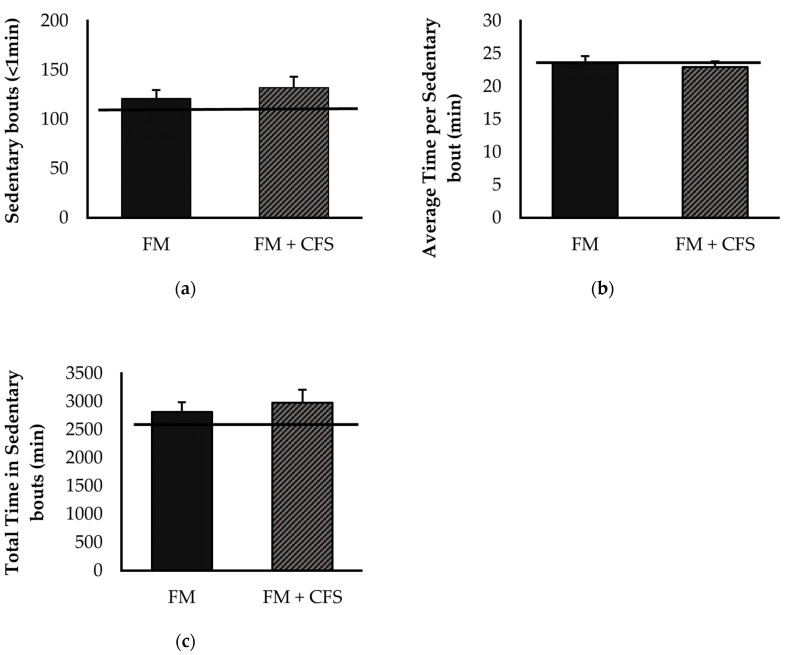
Levels of sedentary lifestyle determined by accelerometry: sedentary bouts (**a**) total time in sedentary bouts (**b**), average time per sedentary bout (**c**) in FM patients with (FM + CFS, *n* = 17) or without (FM, *n* = 17) CFS diagnosis. The horizontal line represents values obtained in the age-matched reference group of “healthy” women. Columns represent the mean ± SEM of independent assays performed in duplicate for each participant. FM: Fibromyalgia, CFS: Chronic Fatigue Syndrome (Mann–Whitney *u*-test).

Overall, no significant differences were found between FM patients with or without CFS codiagnosis in any of the sedentary parameters determined via accelerometry (Figure 6): sedentary bouts (Figure 6a), total time (Figure 6b) and average time (Figure 6c) of sedentary bouts. Although without significant differences, FM patients with and without CFS codiagnosis showed higher sedentary bouts and total time of sedentary bouts than the reference group.

## 4. Discussion

In FM syndrome, the terms woman and pain are deeply related; however, it is also characterized by other disturbances such as sensitivity, pressure allodynia, hyperalgesia, sleep disturbances (nocturnal awakenings, intensification of pain after rest, non-restorative sleep), fatigue, psychological disturbances, etc. [16,36,37]. Despite its increasing prevalence, especially in developed countries, its etiopathogenesis remains unclear and its diagnosis remains a challenge [38,39]. Moreover, it is frequently associated with other syndromes such as CFS, comorbid in up to 80% of cases, where the words woman and fatigue go hand in hand, and where many of the clinical manifestations of CFS are similar to those of FM; therefore, those similar pathophysiological mechanisms are assumed in both processes [16,40,41,42,43]. However, are they so similar? Is there a possibility that there may sometimes be an overlap in the diagnosis of these two syndromes?

It is conceivable that women with a codiagnosis of these two syndromes might be more affected, in terms of quality of life, pain, stress management, and even physical activity, than those without CFS codiagnosis. Indeed, some authors have suggested differences in the association between cognitive performance and pain, noting that comorbidity of CFS and FM should be considered [44].

However, a very recent study conducted by our research group with more than 70 women, with very uniform lifestyles, showed that CFS codiagnosis did not negatively affect the psychological state and already impaired quality of life of patients with FM [16]. In a representative group of these patients, we corroborated in the present investigation that the previous CFS diagnosis in women with FM does not worsen the already altered psychological state of these women, who have higher levels of depression, stress, pain, anxiety, and even fear and anxiety towards to COVID-19, compared to an age-matched reference group of healthy women.

In this context, in the present research, we have sought to determine possible differences in the performance of physical activity and psychoneuroimmunoendocrine biomarkers in women with FM, with and without a previous CFS diagnosis. Fatigue is one of the most commonly used criteria for the diagnosis of FM, and of course, since it is its main symptom, also of CFS. Although Fukuda and coworkers [2] recommend specifically assessing the presence and characteristics of fatigue and other associated symptoms in FM, it is still a highly subjective criterion and is closely associated with other types of previous organic or mental illnesses that cause fatigue or morbid obesity. Because of this fatigue, women with FM tend to be less physically active than healthy people [16,45]. This is equally or more so in the case of CFS, where fatigue becomes the biggest obstacle for these women, not only in physical activity, but also in activities of daily living. The problem lies in the fact that most studies use self-reports or questionnaires to measure activity levels in patients with FM, and although this is a quick and easy method, it is subject to response bias [46,47], as these patients suffer from impaired cognitive function [48]; therefore, self-reported physical activity frequently differs from objectively measured physical activity (e.g., via accelerometry) in patients with FM [49]. In a previous study carried out by our research team, women with FM reported less activity than healthy women, and the CFS codiagnosis did not negatively affect self-reported activity levels despite having reported higher levels of subjective fatigue than women without CFS codiagnosis [16]. However, as we have discussed, subjective measurement of fatigue cannot serve as a substitute for objective monitoring measured with an accelerometer, although it may provide additional information on perceived activity [50]. To objectify levels of physical activity and fatigue, in the present investigation, we have also used the technique of accelerometry, which is very novel in the differential study of FM and CFS, as there are no published studies using this technique to determine levels of physical activity in these two syndromes comparatively. As expected, in the present investigation, we observed that patients with FM have worse levels of physical activity and higher levels of sedentary lifestyle than healthy people; however, we did not find a clear influence of previous CFS diagnosis, even though the latter group again reported higher levels of fatigue. Since objective fatigue was not found to be a differentiating symptom of these two pathologies, we decided to target levels of neuroimmunoendocrine mediators (of inflammation and stress) as potential inducers of objective and perceived pain in both FM patients without previous CFS diagnosis and those with CFS codiagnosis.

The relationship between pain and stress has long been well-known. A lack of control over cortisol suppression after acute stress has been demonstrated in FM, an abnormality that has also been found in individuals with psychiatric disorders [51]. Overall cortisol levels as well as release peaks in individuals with FM are higher than in healthy individuals and individuals with rheumatoid arthritis and, therefore, an altered functioning of HPA axis occurs [3,36,52,53], with this alteration being involved in a multitude of disorders, as proposed by most psychobiotic theories [51]. However, it has been shown in other studies that morning serum [54], saliva [55,56], or hair [56] levels of morning cortisol are lower in individuals with chronic musculoskeletal pain [57], or CFS [54]. Klimas and co-workers [11] propose a loss in the fine regulation of the fight–flight mechanism in CFS, being delayed and attenuated in its amplitude. This hypofunction of the HPA axis in CFS and the lower basal cortisol concentration in these patients has been linked to levels of perceived fatigue [58,59]. Our results are in line with those described by these investigations, as we found that the total group of FM patients has higher cortisol levels than the reference group. However, when we separate the women with a previous CFS diagnosis, we observe that this group has lower cortisol levels than the patients diagnosed only with FM, approaching the values of our reference group of healthy women. It could be hypothesized that the “hyperfunction” of the HPA axis due to FM could be compensated by the hypofunction in CFS. Dysregulation of the HPA axis has also been described by a poor release of DHEA, another endogenous steroid hormone released by the adrenal glands, which may play an etiological role in the maintenance of FM symptomatology [60], as it modulates inflammatory responses through direct inhibition of IL-6 and TNF-α activity [61] and indirectly through promotion of IL-10 [62]. Indeed, decreasing DHEA levels with age have been linked to the development of FM symptomatology throughout life [63]. However, in our results, we found no significant differences in DHEA concentration with respect to the reference group, and no differences between female FM patients with and without a previous CFS diagnosis. Other authors found similar results, proposing that low DHEA levels are more related to age or a postmenopausal state than to the disease itself [60].

Dysregulation of the bidirectional interaction of the cytokine-HPA axis can aggravate inflammatory conditions, and it underlies most autoimmune and inflammatory pathologies, due to a reduced response of the HPA axis to cytokines or to the development of glucocorticoid resistance [64,65]. Thus, disruption of this feedback in FM is associated with a severely dysregulated interaction between the immune/inflammatory and stress responses, particularly mediated by systemic IL-8 and cortisol, but also by other inflammatory cytokines released by monocytes and other stress mediators, such as noradrenaline [3,4,6,66]. In this context, the HPA axis failed to control the increase in pro-inflammatory cytokines [4,5]. Interestingly, the results obtained in the present investigation show this dysregulation in the interaction between the IL-8-mediated inflammatory response and the cortisol-mediated stress response only in patients without a previous CFS diagnosis. These results would suggest that patients with a CFS diagnosis may be overdiagnosed with FM through subjective questionnaires and without the analysis of objective biomarkers, since all previous investigations of our research group showed that women with FM showed elevated levels of IL-8 in relation to healthy women [3]. Our results would not support the idea of Russel and co-workers, who suggested that cytokine levels of IL-1α, IL-6 and IL-8 may serve as robust biomarkers also in the detection of CFS [67].

Additionally, related to the higher levels of pain and depression reported by these patients, we measured noradrenaline and serotonin as objective biomarkers. Indeed, some research has reported that noradrenaline and serotonin reuptake inhibitors are beneficial pharmacological treatments in FM patients [68], probably due to their influence also on the inflammatory response. However, other recent studies have highlighted very poor or no effects [69,70]. The lower systemic serotonin levels in FM patients, already reported in other studies [4,71], and which could explain the lower pain threshold of these patients [72], are clearly consistent with the higher levels of depression perceived by FM patients, both without and with CFS codiagnosis. However, as with perceived levels of fatigue and physical activity, and with neuroimmunoendocrine dysregulation as the mechanism underlying pain, patients with a previous CFS diagnosis showed higher systemic serotonin concentrations than FM patients without CFS, with values very close to those of the control group of healthy women. Again, these results seem to suggest that the CFS diagnosis may induce overdiagnosis of FM through perceived symptoms, which cannot, however, be corroborated using objective biomarkers. This is also true for elevated oxytocin and noradrenaline levels, which are only found to be elevated in FM patients without a CFS codiagnosis. In line with the results obtained for noradrenaline levels, many previous studies reflect higher noradrenaline concentrations in FM patients compared to healthy women [3,4], even without being accompanied by elevated adrenaline levels [73]. Although noradrenaline in healthy conditions can inhibit the release of inflammatory cytokines by immune cells, it has been described that in inflammatory pathologies, it could induce their release [74], a fact that has also been indicated in FM patients [4] and which also explains the results of the present research in the context of neuroimmunoendocrine dysregulation in this disease.

In conclusion, we can say, firstly, that the deterioration in perceived health in patients with FM is corroborated by deterioration in objectively determined physical activity parameters and biomarkers of inflammation and stress. In turn, our results indicate that while perceived health related to fatigue and physical activity capacity, psychological disorders and pain are not affected by a previous CFS diagnosis; physical activity and sedentary lifestyle parameters and objective neuroimmunoendocrine biomarkers related to stress, depression and pain were only manifested in patients without a CFS diagnosis. This suggests a possible overdiagnosis of FM in CFS patients when it is assessed only through perceived symptoms and not with objective immunophysiological parameters. Nevertheless, although the present results can have clinical significance per se, further studies comparing symptoms and objective biomarkers with a big cohort of CFS patients are proposed in order to avoid overdiagnosis of FM.

## Figures and Tables

**Table 1:** Anthropometric characteristics, employment status, and body composition of the participants.

	Reference Group (*n* = 11)	Total FM Patients (*n* = 34)	FM (*n* = 17)	FM + CFS (*n* = 17)	Statistical Significance
Gender (%)	Women (100%)	Women (100%)	Women (100%)	Women (100%)	
Ethnic group (%)	White (100%)	White (100%)	White (100%)	White (100%)	
Duration of FM diagnosed (years)		>2	>2	>2	
Age (years)	55.81 ± 2.08	57.84 ± 1.29	57.20 ± 1.84	58.41 ± 1.85	*p* > 0.05
BMI (kg/m^2^)	24.62 ± 0.83	27.30 ± 0.91	27.42 ± 1.37	27.19 ± 1.25	*p* > 0.05
Employment status					Chi-Square (X^2^) *p* > 0.05 *(*X^2^ > 0.05)
Blue collar workers (%)	18.2	20.6	17.6	23.5	
White collar workers (%)	36.4	11.8	11.8	11.8	
Unemployed (%)	36.4	23.5	29.4	17.6	
Medical leave (%)		23.5	23.5	23.5	
Retired (%)	9.1	20.6	17.6	23.5	
Body composition					
Body fat mass (%)	36.70 ± 1.57	39.61 ± 1.50	38.68 ± 1.54	40.43 ± 2.51	*p* > 0.05
Bone mass (kg)	2.1 ± 0.05	2.11 ± 0.04	2.12 ± 0.05	2.11 ± 0.05	*p* > 0.05
Body water (%)	43.67 ± 1.00	42.55 ± 0.63	42.72 ± 0.97	42.40 ± 0.86	*p* > 0.05
Muscle mass (kg)	39.00 ± 0.80	39.48 ± 1.50	39.64 ± 0.86	39.34 ± 1.06	*p* > 0.05
Visceral fat index	7.31 ± 0.71	9.13 ± 0.47 ^a^	9.10 ± 0.58 ^a^	9.14 ± 0.79 ^a^	*p <* 0.05

Data are expressed as mean ± SEM. RG: Reference Group, FM: Fibromyalgia, CFS: Chronic Fatigue Syndrome, ^a^ *p* < 0.05 with respect to reference group (Student’s *t*-test).

**Table 2:** Psychological state and quality of life.

	RG	Total FM Patients	FM	FM + CFS
Beck’s Depression score	6.00 ± 1.37	20.68 ± 2.21 ^aaa^	17.15 ± 3.12 ^aa^	23.99 ± 2.97 ^aaa^
Perceived Stress score	20.09 ± 2.60	31.03 ± 1.79 ^aaa^	28.60 ± 2.57 ^aa^	33.30 ± 2.41 ^aaa^
State-Trait Anxiety score	15.18 ± 2.29	35.65 ± 2.01 ^aaa^	34.42 ± 2.99 ^aaa^	36.80 ± 2.79 ^aaa^
Healthy Life and Personal Control score	70.72 ± 2.36	66.75 ± 1.90	66.35 ± 1.60	67.13 ± 3.43
Brief Pain Inventory score	1.31 ± 0.48	6.12 ± 0.25 ^aaa^	5.76 ± 0.38 ^aaa^	6.46 ± 0.33 ^aaa^
Brief Fatigue Inventory score	1.46 ± 0.47	6.75 ± 0.33 ^aaa^	6.13 ± 0.53 ^aaa^	7.33 ± 0.36 ^aaa^ *
Fibromyalgia Impact score	3.5 ± 1.48	54.25 ± 2.41 ^aaa^	54.90 ± 3.98 ^aaa^	53.70 ± 3.04 ^aaa^
Fear of COVID-19 score	12.54 ± 1.39	17.62 ± 1.35 ^aa^	17.92 ± 2.18 ^a^	17.33 ± 1.72 ^a^
Coronavirus Anxiety score	0.18 ± 0.12	4.41 ± 1.01 ^aaa^	4.21 ± 1.49 ^aa^	4.60 ± 1.43 ^aa^

Data are expressed as mean ± SEM. RG: Reference Group, FM: Fibromyalgia, CFS: Chronic Fatigue Syndrome, ^a^ *p* < 0.05, ^aa^ *p* < 0.01, ^aaa^ *p* < 0.001 with respect to reference group. * *p* < 0.05 with respect to FM group (Student’s *t*-test).

**Table 3:** Serum levels of neuroimmunoendocrine biomarkers.

	RG	Total FM Patients
IL-8 (pg/mL)	23.04 ± 3.35	26.24 ± 1.91
IL-10 (>LLD)	9.09%	15.62%
Cortisol (pg/mL)	121,848.21 ± 15,010.97	158,072.90 ± 1413.34 ^a^
DHEA (ng/mL)	5.41 ± 0.88	4.48 ± 0.52
Noradrenaline (pg/mL)	168.48 ± 8.42	188.19 ± 6.33 ^a^
Adrenaline (pg/mL)	32.32 ± 4.17	33.31 ± 1.46
Serotonin (ng/mL)	271.07 ± 79.18	156.60 ± 14.29 ^aa^
Oxytocin (pg/mL)	1248.81 ± 120.08	1447.08 ± 93.56

Data are expressed as mean ± SEM. RG: Reference Group, FM: Fibromyalgia, IL-8: Interleukine-8, IL-10: Interleukine-10, LLD: Low Limit of Detection, DHEA: Dehydroepiandrosterone ^a^ *p* < 0.05, ^aa^ *p* < 0.01 with respect to reference group (Student’s *t*-test).

**Table 4:** Physical activity levels and sedentary lifestyle determined via accelerometry.

	RG	Total FM Patients
Metabolic rate (METs)	1.61 ± 0.09	1.46 ± 0.04
Activity bouts (<1 min)	95.90 ± 13.36	67.27 ± 6.33 ^a^
Total Time in Activity bouts (min)	1968.00 ± 334.77	1190.51 ± 135.44 ^a^
Average Time per Activity bout (min)	19.33 ± 1.18	16.68 ± 0.73 ^a^
Steps count (nº steps)	109,631.00 ± 9800.09	76,069.51 ± 4841.51 ^aa^
Sedentary bouts (<1 min)	114.50 ± 8.98	126.96 ± 6.96
Total Time in Sedentary bouts (min)	2567.00 ± 131.44	2902.41 ± 142.54 ^a^
Average Time per Sedentary bout (min)	23.07 ± 1.21	23.28 ± 0.66

Data are expressed as mean ± SEM. RG: Reference Group, FM: Fibromyalgia, MET: Metabolic Equivalent of Task. ^a^ *p* < 0.05, ^aa^ *p* < 0.01 with respect to reference group. (Student’s *t*-test).

## Data Availability

The raw data supporting the conclusions of the manuscript will be made available by the authors, without undue reservation, to any qualified researcher.
